# Synergistic self-assembly and crosslinking yield a durable, bioactive, and injectable recombinant collagen implant for photoaging therapy

**DOI:** 10.1093/rb/rbag026

**Published:** 2026-03-02

**Authors:** Nannan Wei, Yuchen Zhang, Xinyu Tian, Linyan Yao, Jianxi Xiao

**Affiliations:** State Key Laboratory of Natural Product Chemistry, College of Chemistry and Chemical Engineering, Lanzhou University, Lanzhou 730000, PR China; Gansu Engineering Research Center of Medical Collagen, Lanzhou 730000, PR China; State Key Laboratory of Natural Product Chemistry, College of Chemistry and Chemical Engineering, Lanzhou University, Lanzhou 730000, PR China; Gansu Engineering Research Center of Medical Collagen, Lanzhou 730000, PR China; State Key Laboratory of Natural Product Chemistry, College of Chemistry and Chemical Engineering, Lanzhou University, Lanzhou 730000, PR China; Gansu Engineering Research Center of Medical Collagen, Lanzhou 730000, PR China; Gansu Engineering Research Center of Medical Collagen, Lanzhou 730000, PR China; State Key Laboratory of Animal Disease Control and Prevention, College of Veterinary Medicine, Lanzhou University, Lanzhou 730000, PR China; State Key Laboratory of Natural Product Chemistry, College of Chemistry and Chemical Engineering, Lanzhou University, Lanzhou 730000, PR China; Gansu Engineering Research Center of Medical Collagen, Lanzhou 730000, PR China

**Keywords:** self-assembled nanofibers, BDDE-crosslinked recombinant collagen, extracellular matrix regeneration, skin rejuvenation

## Abstract

Sustained ultraviolet (UV) irradiation upregulates matrix metalloproteinases (MMPs) activity, exacerbates collagen degradation, and triggers inflammatory signaling, leading to extracellular matrix (ECM) disorganization and skin photoaging. While animal-derived collagens are widely used in clinical applications, their immunogenicity and batch variability limit safety and consistency. Recombinant collagen provides a safer and more controllable alternative. However, poor fibrillogenesis, limited structural stability, and rapid enzymatic degradation hinder long-term performance. Here, we developed an injectable recombinant collagen nanofiber implant (B-SARCI) through the synergistic integration of molecular self-assembly and mild 1,4-butanediol diglycidyl ether (BDDE) crosslinking. This dual strategy preserves excellent injectability while simultaneously enhancing thermal stability, and enzymatic resistance. B-SARCI improved fibroblast attachment and proliferation, promoted migration, and facilitated differentiation *in vitro*. In a murine UV-induced photoaging model, B-SARCI restored dermal density, enhanced barrier function, and reduced transepidermal water loss (TEWL). Transcriptomic and histological analyses revealed upregulation of Col1α1 and Col3α1, suppression of MMP2, MMP3, and MMP9, and modulation of the JAK-STAT pathway via SOCS3 induction and IL-6 downregulation. Together, these findings demonstrate that B-SARCI re-establishes ECM homeostasis and attenuates inflammation, representing a durable, bioactive, and clinically translatable recombinant collagen implant for photoaged skin repair.

## Graphical Abstract

**Figure:**
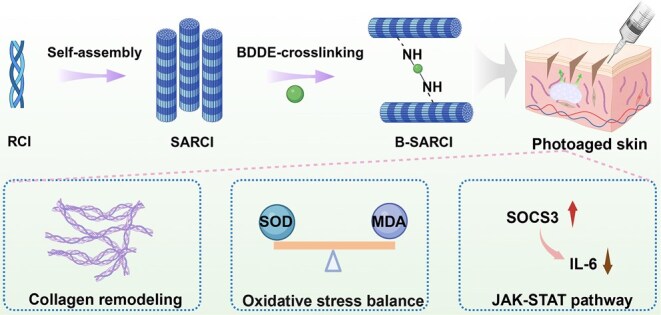

## Introduction

Skin aging represents an unavoidable physiological progression arising from the combined effects of endogenous aging and external environmental exposures [1, 2]. Among these, chronic ultraviolet (UV) exposure is widely recognized as the predominant driver of photoaging, leading to collagen degradation, activation of matrix metalloproteinases (MMPs), and disruption of dermal extracellular matrix (ECM) homeostasis [3–6]. The persistent oxidative and inflammatory stress associated with UV radiation not only accelerates tissue degeneration but also increases susceptibility to actinic keratosis, basal cell carcinoma, and other skin malignancies [7]. Consequently, developing effective biomaterial-based strategies to repair photoaged skin and restore dermal functionality remains a critical clinical need.

Collagen is the dominant protein within the dermal ECM, and its characteristic triple helix drives the formation of multiscale fibrillar networks, providing tensile strength and mechanical resilience to the skin [8–11]. Owing to its excellent biocompatibility and biological relevance, collagen is widely used as both a scaffold and an injectable filler in aesthetic and regenerative applications [12, 13]. However, traditional animal-derived collagen suffers from intrinsic limitations, such as potential immunogenicity, the risk of pathogen transmission, and significant batch-to-batch variability, which collectively constrain its clinical translation [14]. These challenges have catalyzed the development of collagen biomaterials with higher purity, controllable properties, and improved biosafety.

Recombinant collagen is generated by genetic engineering and serves as a pathogen-free option with improved immunological safety compared with animal-sourced materials [15, 16]. Nevertheless, most recombinant collagens exhibit poor fibrillogenesis and rapid enzymatic degradation *in vivo*, resulting in insufficient mechanical stability and short residence time within tissue microenvironments [17]. Hence, the design of recombinant collagen implants that simultaneously achieve molecular-level self-assembly, structural durability, and injectability remains a major challenge in advancing functional anti-aging therapeutics.

**Figure 1:**
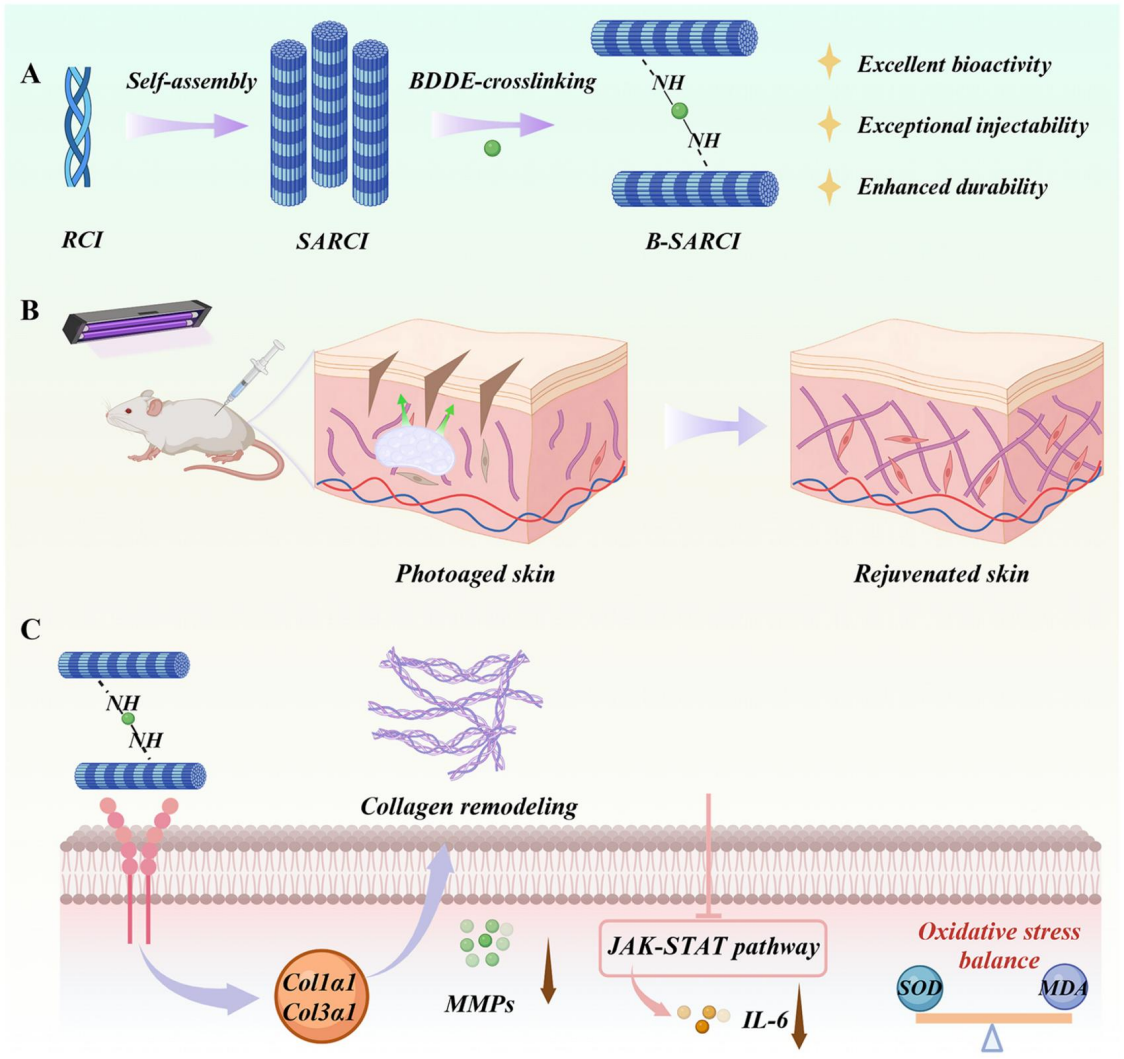
Schematic representation of the process used to prepare the injectable recombinant type I collagen nanofiber implant (B-SARCI). The construct is prepared through sequential molecular self-assembly followed by BDDE crosslinking, yielding a structurally stable and syringe-injectable nanofiber network. The B-SARCI implant restores ECM homeostasis, enhances dermal density, and repairs photoaged skin by reconstructing collagen architecture and attenuating inflammation. Created with BioRender.com.

Herein, we report an injectable recombinant type I collagen nanofiber implant (B-SARCI) developed via a synergistic strategy that couples self-assembly with 1,4-butanediol diglycidyl ether (BDDE) crosslinking (Figure 1). This dual mechanism enables B-SARCI to achieve excellent injectability, superior thermostability, and remarkable enzymatic resistance. *In vitro*, B-SARCI promotes fibroblast adhesion, migration, proliferation, and differentiation. In a mouse model of photoaging induced by UV irradiation, B-SARCI supports ECM homeostasis, enhances dermal density, and reestablishes skin barrier function. Collectively, this work presents a durable, bioactive, and injectable recombinant collagen implant with strong potential for aesthetic and regenerative dermatology.

## Materials and methods

### Preparation and characterization of recombinant type I collagen

#### Expression of recombinant type I collagen

Following synthesis of the recombinant type I collagen (RCI) gene and insertion into the pCold III plasmid, the construct was introduced into the *E. coli* BL21 (DE3) strain. The RCI amino acid sequence is listed in Table S1. 20 μL of glycerol stock was added to LB medium (200 mL) containing kanamycin sulfate (50 mg/L, Aladdin Biochem Technology, China) and incubation at 37°C with orbital shaking at 220 rpm. The initial culture was used to inoculate 1 L LB (2% *v/v* inoculum) and grown until the optical density (OD_600_) reached 1.2–2.0. Protein expression was subsequently induced by adding 1 mM IPTG (Biotopped Life Sciences, China) and continuing incubation at 25°C for a period of 16–20 h. The resulting biomass was harvested via centrifugation and then kept at −80°C.

The bacterial pellets were resuspended in phosphate buffer (20 mM, pH 7.4) containing NaCl (500 mM, Chengdu Kelong Chemical, China) and imidazole (20 mM, Biotopped Life Sciences, China). To clarify the lysate, centrifugation was conducted at 10 000 rpm for 30 min (4°C). Clarified supernatant was subsequently applied to a Ni-affinity column for affinity capture. Target proteins were recovered using a linear imidazole gradient ranging from 20 to 500 mM. Thrombin (Sangon Biotechnology, China) was employed to cleave the foldon tag and the final product was dialyzed against ultrapure water using molecular-weight-selective membranes (8–14 kDa).

#### Sodium dodecyl sulfate-polyacrylamide gel electrophoresis (SDS-PAGE) assessment

SDS-PAGE was carried out on a mini vertical electrophoresis system (DYCZ-24DN, Beijing Liuyi Biotech., China) with a 5% stacking gel and a 12% resolving gel. RCI (1.0 mg/mL) was combined with loading buffer at a 2:1 (*v/v*) ratio, and 10 μL was loaded per well. A protein ladder ranging from 10 to 180 kDa (Thermo Scientific, USA) was used as a reference standard.

#### Circular dichroism (CD)

CD measurements were performed on a JASCO spectropolarimeter (JASCO J-1500, Japan) equipped with a Peltier device. Solubilization of RCI (1.0 mg/mL) was achieved in 10 mM sodium phosphate (pH 7.4), followed by a 24-h low-temperature incubation (4°C). CD spectra were acquired in a quartz cuvette (path length, 1.0 mm) over 190–260 nm.

### Preparation of self-assembled recombinant type I collagen nanofibers

The purified RCI was dissolved in phosphate buffer (50 mM, pH 7.4) and allowed to self-assemble at 25°C for 24 h. The suspension was centrifuged at 10000 rpm for 10 min at 4°C. The precipitate fraction was then harvested as self-assembled recombinant type I collagen nanofibers (SARCI).

### Preparation of BDDE-crosslinked SARCI (B-SARCI)

SARCI was redispersed in phosphate buffer (50 mM, pH 7.4) to achieve a final concentration of 50 mg/mL. BDDE (Aladdin Biochem Technology, China) was then added at four predefined concentrations (0.5%, 1%, 2%, and 5%, *v/v*) and mixed thoroughly to ensure uniform mixing. The mixture was incubated at 25°C for 24 h to complete crosslinking, then centrifuged (10 000 rpm, 10 min, 4°C). The precipitate was washed with ultrapure water (3–5 cycles) to remove unreacted BDDE. The residual BDDE content in B-SARCI was determined by fluorometric analysis utilizing a Tecan Infinite F200/M200 microplate reader (λex = 370 nm, λem = 430 nm) [18].

### Characterization of B-SARCI

#### Degree of crosslinking

The 2,4,6-trinitrobenzenesulfonic acid (TNBS, Sigma-Aldrich, China) assay was used to quantify the degree of crosslinking of SARCI treated with different concentrations of BDDE. Accurately weighed 11 mg of SARCI (denoted as A_1_) and BDDE-crosslinked (denoted as A_2_) lyophilized collagen nanofiber samples were each mixed with 1 mL of 4% NaHCO_3_ solution and 0.5% TNBS solution. A blank control group (A_0_) was included to correct for background absorbance: 11 mg of SARCI was initially mixed with 3 mL of 6 mol/L HCl, followed by sequential addition of 1 mL of 4% NaHCO_3_ and 0.5% TNBS solution.

Initial incubation of all samples was conducted at 40°C with constant agitation (220 rpm) for 4 h. To terminate the reaction, 3 mL of 6 mol/L HCl introduced into the A_1_ and A_2_ samples, followed by a secondary heating step at 60°C for 1.5 h. A 1 mL aliquot of the resulting mixture was then diluted with 4 mL of ultrapure water. To eliminate residual TNBS, 2 mL of this diluted solution underwent liquid-liquid extraction three times using 8 mL of ether. Finally, the aqueous phase (1 mL) was further diluted with 4 mL of water, and its optical density was recorded at 345 nm. The crosslinking degree was calculated using Equation (1):


(1) $$\text{Degree}\;\text{of}\;\text{crosslinking}\;(\%)=(1-\frac{A_{2}-A_{0}}{A_{1}-A_{0}})\times 100\%\;$$

#### Fourier transform infrared (FTIR) spectroscopy

Lyophilized SARCI and B-SARCI samples (2 mg) were accurately weighed and mixed with potassium bromide (KBr) (200 mg). The resulting fine powder was compressed into translucent disks (10 mm diameter) using a hydraulic tablet press. Spectral acquisition was performed on a Bruker Tensor 27 spectrometer (Germany) across a wavenumber range of 4000–400 cm^−1^.

#### Differential scanning calorimetry (DSC)

Lyophilized SARCI and B-SARCI samples (5–10 mg) were placed in sealed aluminum crucibles and subjected to DSC analysis (Mettler Toledo DSC3+). The thermal behavior was monitored during a heating cycle from 25 to 200°C at a constant rate of 10°C/min, with 50 mL/min of nitrogen flow providing an inert environment.

#### Scanning electron microscope (SEM)

B-SARCI samples were affixed onto sample holders using conductive adhesive, ensuring a flat surface and good contact. To ensure adequate surface conductivity, the specimens were subjected to gold sputtering for a duration of 30 s. After pretreatment, the samples were placed in a SEM (Hitachi S-4800, Hitachi, Japan), and microstructural images were captured under appropriate accelerating voltage.

#### Injectability

The evaluation of injection force was performed by extruding SARCI and B-SARCI through 26G needles using a Bosin Tech TA.20 texture analyzer (China). The plunger speed was maintained at 30 mm/min, and the resistance to injection was calculated from the real-time force-displacement profiles.

#### Collagenase degradation

*In vitro* enzymatic stability was evaluated by immersing 10 mg of lyophilized SARCI and B-SARCI into a collagenase-active solution (5 U/mL) prepared in 50 mM TES buffer containing 1 mM CaCl_2_ (pH 7.4). To ensure uniform digestion, the samples were agitated at 120 rpm in a controlled 37°C environment for the duration of the study. After incubation for a predetermined duration under set conditions, the residual samples were lyophilized once more and weighed to determine mass loss. The extent of mass loss, expressed as the degradation rate (DR, %), was quantified using the following equation:


(2) $$\text{DR}\;(\%)=\frac{m_{0}-m_{t}}{m_{t}}\times 100\%\;$$

where m_0_ and m_t_ are the sample masses before and after the enzymatic treatment, respectively.

#### Endotoxin content

The endotoxin level was determined using an established method based on the dynamic turbidimetric Limulus amebocyte lysate (LAL) assay [19]. Briefly, all glassware was depyrogenated (250°C, ≥2 h), and B-SARCI samples were diluted with endotoxin-free water. The determination of endotoxin levels was carried out using a BET-48G detector (Tianda Tianfa, China). The assay involved monitoring the reaction mixture (LAL reagent and sample) at 37°C and recording the onset time required for the optical density at 405 nm to reach a threshold of 0.02.

#### Residual DNA content

The quantification of residual DNA was carried out utilizing the Quant-iT PicoGreen dsDNA assay kit. In brief, B-SARCI samples were diluted in 1× TE buffer. Concurrently, a calibration curve was established using the provided Lambda DNA standards. A fresh working solution of the PicoGreen reagent was prepared by dilution in 1× TE buffer, ensuring protection from light exposure at all stages. The assay involved combining aliquots of the test samples or standards with the reagent in a black 96-well microplate, followed by a 5-min incubation at ambient temperature. Subsequent fluorescence detection was performed on a Tecan Infinite F200/M200 reader (λex = 480 nm, λem = 520 nm), the final dsDNA concentration was determined using the calibration curve.

### *In vitro* cell assays for B-SARCI

#### Cell culture

Human foreskin fibroblasts (HFF-1) cells, originating from the Cell Bank of the Chinese Academy of Sciences (Shanghai), were maintained under standard conditions. The growth medium consisted of DMEM enriched with 15% FBS alongside 1% penicillin-streptomycin.

#### Cell adhesion analysis

To evaluate cell-material interactions, untreated 6-well plates were surface-modified by applying SARCI, B-SARCI, or G-SARCI solutions (10 mg/mL in PBS) and incubating them overnight at 4°C. As a non-adhesive reference, parallel wells were treated with 1% heat-denatured bovine serum albumin (BSA). HFF-1 cells were subsequently inoculated into the prepared wells at a density of 2 × 10^5^ cells/mL (500 μL per well). Following a 12-h attachment period, cellular spreading and morphology were visualized utilizing an Olympus IX53 inverted microscope (Japan).

#### Cell migration assay

For the migration assay, HFF-1 cells (2 × 10^5^ cells/mL) were cultured in 6-well plates in complete medium. A linear gap was introduced into the uniform cell carpet after it reached ∼90% density. This was accomplished by dragging a 20 μL micropipette tip across the well to simulate a wound site. Detached cells were rinsed away with PBS, and the remaining adherent cells were exposed to the test materials (SARCI, B-SARCI, or G-SARCI at 10 mg/mL) or a blank control (DMEM only). After a 24-h incubation, gap closure was visualized utilizing an Olympus IX53 microscope. Quantitative analysis was performed on at least three independent fields using ImageJ, calculated as follows:


(3) $$\text{Migration}\;(\%)=\frac{A_{0}-A_{24}}{A_{0}}\times 100\%\;$$

where A_0_ and A_24_ denote the scratch area measured at 0 and 24 h, respectively.

#### Cell proliferation assay

HFF-1 cells (100 μL/well, 1 × 10^5^ cells/mL) were inoculated into 96-well plates and maintained in complete medium for 24 h. After incubation, the culture medium was replaced with DMEM containing SARCI, B-SARCI, or G-SARCI at 10 mg/mL, DMEM alone was used for the blank group. Cells were cultured for a duration of 1, 3, or 5 days, during which the treatment medium was replenished every 48 h. To assess cell viability at the designated time points, Cell Counting Kit-8 (CCK-8) solution was added to each well and incubated at 37°C for 1 h. The optical density was quantified at 450 nm using a Tecan Infinite F200/M200 microplate reader. All assays were conducted in triplicate, and the entire experiment was performed independently at least three times.

#### Live/dead staining

Following a 24 h adhesion period in confocal dishes (1 × 10^5^ cells/mL), cells were exposed to DMEM containing 10 mg/mL of SARCI, B-SARCI, or G-SARCI, using DMEM alone as a blank group. The incubation continued for 1, 3, and 5 days, with fresh medium replacement occurring every 2 days. Cell viability across different time points was determined using Live/Dead kit (Solarbio, CA1630), with high-resolution imaging conducted on an Olympus FV3000 confocal microscope.

#### Visualization of cytoskeletal architecture

After seeding on pre-coated SARCI, B-SARCI, or G-SARCI surfaces (prepared by coating untreated 6-well plates with 10 mg/mL formulations in PBS overnight at 4°C), HFF-1 cells (1 × 10^5^ cells/mL) were cultured in DMEM for 5 days. Cells were first fixed in 4% paraformaldehyde treatment (10 min). Subsequently, a 5-min treatment with Triton X-100 (0.1%) was applied for permeabilization, and nonspecific binding was suppressed by incubation in 1% BSA for 30 min. For visualization, F-actin filaments were probed with TRITC-conjugated phalloidin (100 nM) for 1 h, while nuclear topography was defined by a 20-min incubation with Hoechst 33258 (5 μg/mL). The resulting fluorescent signals were captured using an Olympus FV3000 confocal system.

#### Gene expression analysis

HFF-1 cells (1 × 10^5^ cells/mL) were inoculated into 6-well plates and cultured in DMEM (blank group) or DMEM containing SARCI, B-SARCI, or G-SARCI (10 mg/mL) for 7 days, with the corresponding treatment medium refreshed every 2 days. Following RNA extraction (SM129-02, Seven), cDNA was synthesized via reverse-transcription using a PrimeScript RT reagent kit (RR047A, Takara). RT-qPCR was set up with TB Green Premix Ex Taq II (RR820A, Takara) and performed on an Mx3005P platform (Agilent). Using GAPDH as the internal reference, the expression of *α-SMA*, *Collagen I* and *Collagen III* was quantified via the 2^–ΔΔCt^ calculation. Specific primers designed for the quantification of target gene are detailed in Table 1.

**Table 1:** RT-qPCR Primer sets designed for target gene quantification.

Target gene	Forward primer (5′-3′)	Reverse primer (5′-3′)
*α-SMA*	CTGCTGAGCGTGAGATTGTC	CTCAAGGGAGGATGAGGATG
*Collagen I*	TTCACCTACAGCACGCTTGT	TTGGGATGGAGGGAGTTTAC
*Collagen III*	GGTCACTTTCACTGGTTGACGA	TTGAATATCAAACACGCAAGGC

### *In vivo* study of B-SARCI on UV-mediated photoaging

#### Model induction and treatment schedule

All procedures involving laboratory animals adhered to the welfare guidelines established by the Chemistry and Chemical Engineering Ethics Committee at Lanzhou Universit (Approval No. G20). Female Kunming mice, weighing 20–25 g and aged 6–8 weeks, were obtained from the Lanzhou University animal center. Prior to the experiments, the animals were maintained for a 7-day period under standardized laboratory settings. Following dorsal hair removal, the mice randomized into five experimental groups (*n* = 18 each): (i) healthy group (blank), (ii) UV-exposed pathological models, (iii) SARCI group, (iv) B-SARCI group, and (v) G-SARCI group. Mice in the SARCI, B-SARCI, and G-SARCI groups received a 100 μL injection of the corresponding formulation (80 mg/mL) into the dorsal space beneath the dermis. Following injection, the injection sites were monitored daily for local reactions (e.g. ulceration and signs of infection), and no abnormalities were observed.

To replicate the synergistic effects of natural solar radiation, photoaging was triggered through a dual-spectrum (UVA and UVB) irradiation strategy, accounting for the distinct dermal and epidermal penetration depths of these wavelengths [20, 21]. Following depilation and antiseptic cleaning of the back, UVA (300–420 nm, peak at 340 nm; T8, 30 W; YiXian, China) and UVB (280–320 nm, peak at 313 nm; T8, 30 W; YiXian, China) was applied to the exposed dorsal skin at a fixed 20 cm working distance to establish the photoaging model. During exposure, mice were individually restrained in a well-ventilated restrainer to minimize movement and maintain a consistent irradiation setup. The determined MED values were 0.29 and 6.71 mJ/cm^2^ for UVA and UVB, respectively. Mice then underwent a 6-week dose-escalation irradiation regimen (Table 2). This schedule resulted in a final accumulated exposure of 20.30 J/cm^2^ for UVA and 0.47 J/cm^2^ for UVB.

**Table 2:** UVA/UVB Doses used for photoaging induction.

Week	Frequency	Dose
1	Daily	1 MED
2	Daily	2 MED
3-4	Every other day	3 MED
5-6	Every other day	4 MED

#### Biocompatibility of B-SARCI *in vivo*

Following the implantation procedure, animals were euthanized at designated time points to facilitate the excision of skin specimens. Tissue samples were subjected to fixation in 4% paraformaldehyde, followed by embedding and sectioning to prepare for histological examination. Both H&E and immunohistochemical techniques were utilized for histological profiling, with a focus on quantifying markers of interest such as MPO, iNOS, and CD206. At the 4-week post-implantation, subcutaneous tissues were excised to evaluate the local inflammatory profile. Specific ELISA kits were employed to measure the protein levels of interleukin (IL)-1β, alongside the regulatory cytokines IL-4 and IL-10. Furthermore, to investigate potential systemic biosafety, the heart, spleen, liver, kidneys, and lungs were excised after 6 weeks of treatment. The collected tissues were subjected to H&E staining.

#### Noninvasive evaluation and histological examination

The DermaLab Combo system was employed to monitor skin changes. The assessment protocol included high-frequency ultrasound imaging and videoscopic examination, as well as the quantification of skin hydration and transepidermal water loss (TEWL). Following euthanasia at designated endpoints, dorsal skin tissues were collected and immersion-fixed in 4% paraformaldehyde. The samples were subsequently embedded in paraffin wax and sliced into 5 μm sections. Sections were processed for H&E staining, as well as Masson’s trichrome and picrosirius red staining. Histological evaluation of skin architecture and collagen remodeling was conducted using a light microscope (Olympus BX63, Japan).

The peptide probe (FAM-GOP-14) was employed to identify denatured collagen within the skin tissues, followed by imaging on an Olympus FV3000 confocal laser scanning microscope. For the assessment of oxidative stress, commercial assay kits were utilized to measure the activity of superoxide dismutase (SOD) and the levels of malondialdehyde (MDA), in strict compliance with the manufacturers’ instructions.

#### Transcriptomic profiling

Total RNA was isolated from the skin tissues of mice in the B-SARCI and model groups. The integrity of the extracted RNA was verified using an Agilent 2100 Bioanalyzer. Subsequently, samples meeting quality standards underwent library preparation and were sequenced via the Illumina platform by Novogene (Beijing, China). Differentially expressed genes (DEGs) were identified by applying significance thresholds of |log_2_(fold change)| ≥ 0.58 and *P* values < 0.05. Gene Ontology (GO) and Kyoto Encyclopedia of Genes and Genomes (KEGG) pathway enrichment were used to summarize functional categories (BP, CC, and MF) and to highlight the pathways most associated with the gene set.

#### Immunofluorescence-based staining

Cutaneous sections were processed by deparaffinization and rehydration in graded ethanol, followed by heat-mediated antigen retrieval. Sections were permeabilized with Triton X-100 (0.5%) and blocked using 5% goat serum. Subsequently, the samples were treated with anti-Col1α1, anti-Col3α1, anti-MMP2, anti-IL-6, and anti-SOCS3 primary antibodies overnight at 4°C. Visualization was achieved by incubating the samples with fluorescent secondary antibodies for 1 h at room temperature. Nuclear staining was performed using DAPI, followed by mounting with an antifade solution to preserve fluorescence. In addition, CD68 expression was visualized via immunofluorescence in skin sections collected at weeks 2, 4, and 6 to assess macrophage infiltration dynamics. Image acquisition was performed using an Olympus FV3000 confocal laser scanning microscope.

### Statistical analysis

Data are reported as mean ± SD. *In vitro* experiments were performed in triplicate at minimum and repeated in ≥3 independent runs. Group differences were evaluated by one-way ANOVA with Tukey’s *post hoc* analysis. Significance thresholds were set at *P < 0.05, **P < 0.01, and ***P < 0.001.

## Results and discussion

### Characterization of recombinant type I collagen

To assess the purity and determine the apparent molecular weight of recombinant type I collagen (RCI), sodium dodecyl sulfate-polyacrylamide gel electrophoresis (SDS-PAGE) was conducted. RCI migrated as a single predominant band with no obvious additional bands, indicating a high degree of purity (Figure S1A).

The triple-helical conformation of RCI was analyzed by CD spectroscopy. The spectrum displayed a positive band near 220 nm and a negative band around 198 nm, consistent with the characteristic signature of collagen triple helices (Figure S1B).

### Characterization of B-SARCI

**Figure 2:**
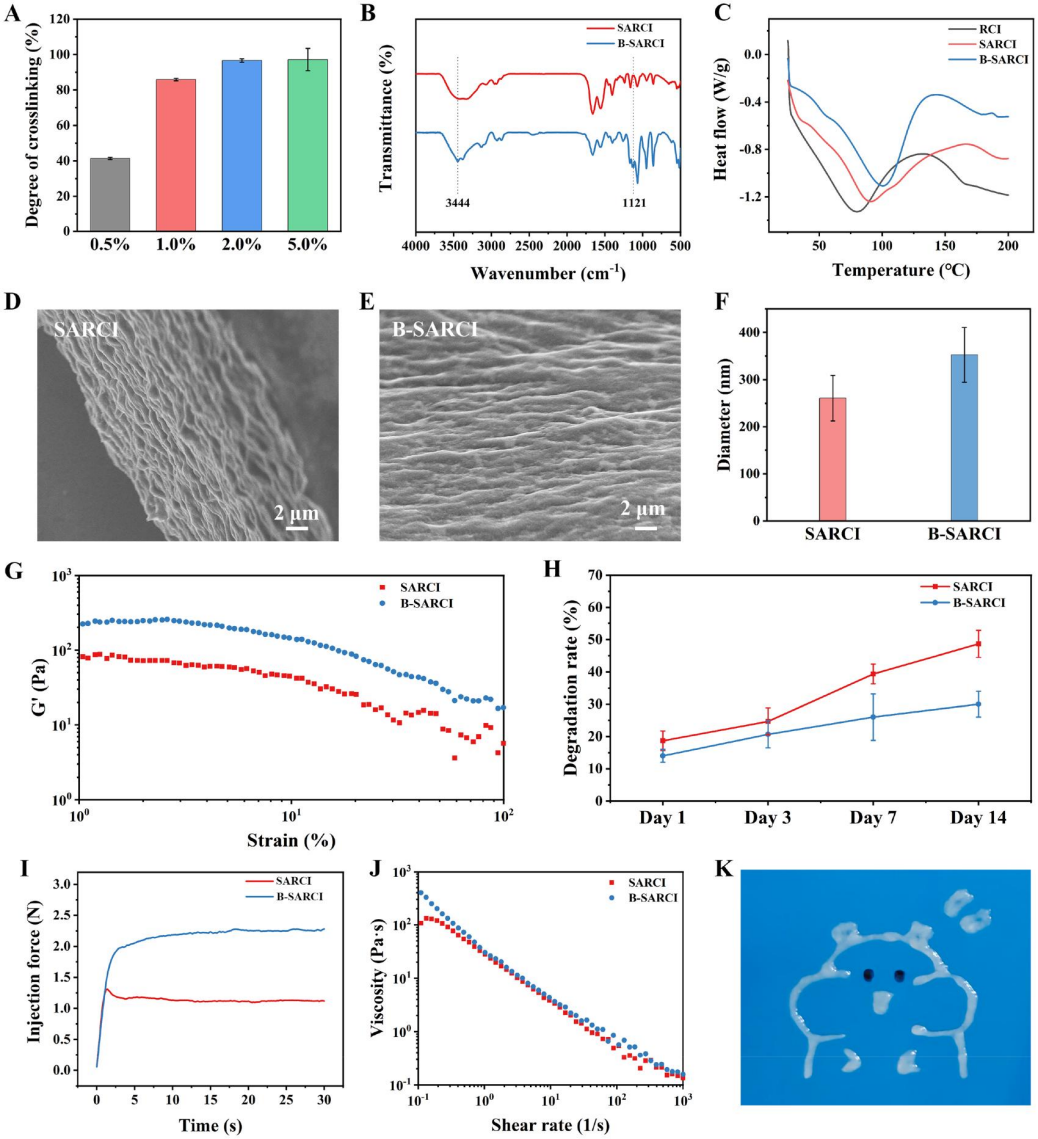
Characterization of B-SARCI. (**A**) Degree of crosslinking quantified by the TNBS assay. (**B**) FTIR spectra of SARCI and B-SARCI. (**C**) DSC profiles of SARCI and B-SARCI. Representative SEM images of (**D**) SARCI and (**E**) B-SARCI. (**F**) Analysis of nanofiber diameters. (**G**) Storage modulus (G') of SARCI and B-SARCI. (**H**) Collagenase-induced degradation behavior of SARCI and B-SARCI. (**I**) Force-displacement curves evaluating injectability through a syringe needle. (**J**) The viscosity of SARCI and B-SARCI as a function of shear rate. (**K**) Photographic demonstration of smooth extrusion of B-SARCI under constant force.

The crosslinking degree of recombinant type I collagen nanofibers treated with varying BDDE concentrations was determined using the 2,4,6-trinitrobenzenesulfonic acid (TNBS) assay (Figure 2A). The crosslinking degree increased progressively from 41.3% at 0.5% BDDE to 85.8, 96.6, and 97.7% at 1, 2, and 5%, respectively, indicating a concentration-dependent enhancement in crosslinking efficiency. Considering that further elevation from 2 to 5% BDDE produced only a marginal increase, 2% BDDE was used in the following experiments to fabricate crosslinked self-assembled recombinant type I collagen nanofibers (B-SARCI).

The effective integration of BDDE crosslinks within the self-assembled collagen nanofibers was validated by FTIR spectroscopy (Figure 2B). The native collagen spectrum exhibited signature vibrational modes: the Amide A and B bands were observed at 3375 and 3074 cm^−1^, respectively. Additionally, the characteristic signals for Amides I, II, and III were centered at 1660, 1556, and 1240 cm^−1^, respectively. After BDDE treatment, additional absorption peaks appeared at 1119 cm^−1^ (C-O stretching) and 3444 cm^−1^ (Amide A), indicating the formation of ether bonds between BDDE epoxy groups and collagen amino residues, thus verifying the establishment of covalent crosslinking within the nanofibrous network.

Thermal stability was evaluated by DSC (Figure 2C). The denaturation temperature was 80.0°C for RCI and increased to 89.8°C for SARCI, reflecting enhanced thermal stability after assembly. Following BDDE crosslinking, the denaturation temperature further increased to 101.5°C for B-SARCI, indicating that crosslinking effectively enhanced the overall conformational rigidity. Scanning electron microscopy (SEM) revealed fibrillar nanostructures in both SARCI and B-SARCI (Figure 2D and E). Compared with SARCI, B-SARCI preserved a dense and uniformly distributed nanofibrillar architecture, suggesting controlled interfibrillar crosslinking without structural collapse. Furthermore, BDDE crosslinking significantly increased the average nanofiber diameter from 260.7 ± 48.2 to 352.4 ± 58.1 nm (Figure 2F). While these results support nanofiber formation and its stabilization by crosslinking, the specific sequence and structural determinants governing fibril formation and their contributions to the assembly process remain to be clarified.

Oscillatory shear rheology was performed to assess the viscoelastic properties of B-SARCI (Figure 2G). The storage modulus (G') of B-SARCI is consistently higher than that of SARCI, indicating increased network stiffness after crosslinking.

Proteolytic stability was examined by collagenase digestion (Figure 2H). Both materials underwent gradual enzymatic degradation. However, after 14 days, B-SARCI retained approximately 70.0% of its initial mass compared with 51.3% for SARCI, confirming that BDDE crosslinking markedly enhanced enzymatic resistance-a critical property for maintaining implant integrity *in vivo*.

The injectability of B-SARCI was evaluated by measuring the extrusion force required to pass the material through a 26G syringe needle (Figure 2I). The required force increased slightly from 1.1 N for SARCI to 2.2 N for B-SARCI, reflecting greater mechanical robustness after crosslinking. Importantly, this force remained well below the clinical threshold of 10 N, demonstrating excellent syringeability and smooth extrusion behavior [22, 23]. The viscosity-shear rate measurements further demonstrated that B-SARCI exhibits stable shear-thinning behavior, supporting its suitability for injectability (Figure 2J). The uniform flow under constant load qualitatively demonstrates the extrudability of B-SARCI through a syringe needle under the tested conditions (Figure 2K). Collectively, these findings indicate that mild BDDE crosslinking preserves the nanoscale architecture of self-assembled recombinant collagen while significantly enhancing its thermal, enzymatic, and mechanical stability, thereby yielding an injectable yet structurally durable implant suitable for long-term dermal repair.

Quality-control assessment was conducted on the B-SARCI to verify critical quality control parameters relevant to product safety. Residual BDDE was quantified by a fluorescence-based assay to evaluate potential biocompatibility risks associated with crosslinker residues (Table S2). The residual BDDE content was 1.35 ± 0.24 ppm, which is below the specified limit of 2 ppm [24]. The bacterial endotoxin level was 0.28 ± 0.02 EU/mL, and the residual DNA content was 4.27 ± 0.58 ng/mL. Both values are below commonly referenced acceptance criteria [25, 26].

Glutaraldehyde-crosslinked SARCI (G-SARCI) was prepared as a reference crosslinking control and characterized in terms of crosslinking degree, thermal stability, nanofiber morphology, and extrusion force (Figure S2). At 2% (*v/v*) glutaraldehyde, G-SARCI exhibited a crosslinking degree of 96.3%, comparable to that of SARCI crosslinked with 2% (*v/v*) BDDE (96.6%). Under these conditions, G-SARCI showed a denaturation temperature of 101.0°C, a fiber diameter of 362.7 ± 73.4 nm, and an extrusion force of ∼3 N, indicating physicochemical properties broadly comparable to those of B-SARCI. This formulation was used as the crosslinking control in subsequent *in vitro* and *in vivo* evaluations.

### Evaluation of cell behaviors on B-SARCI

**Figure 3:**
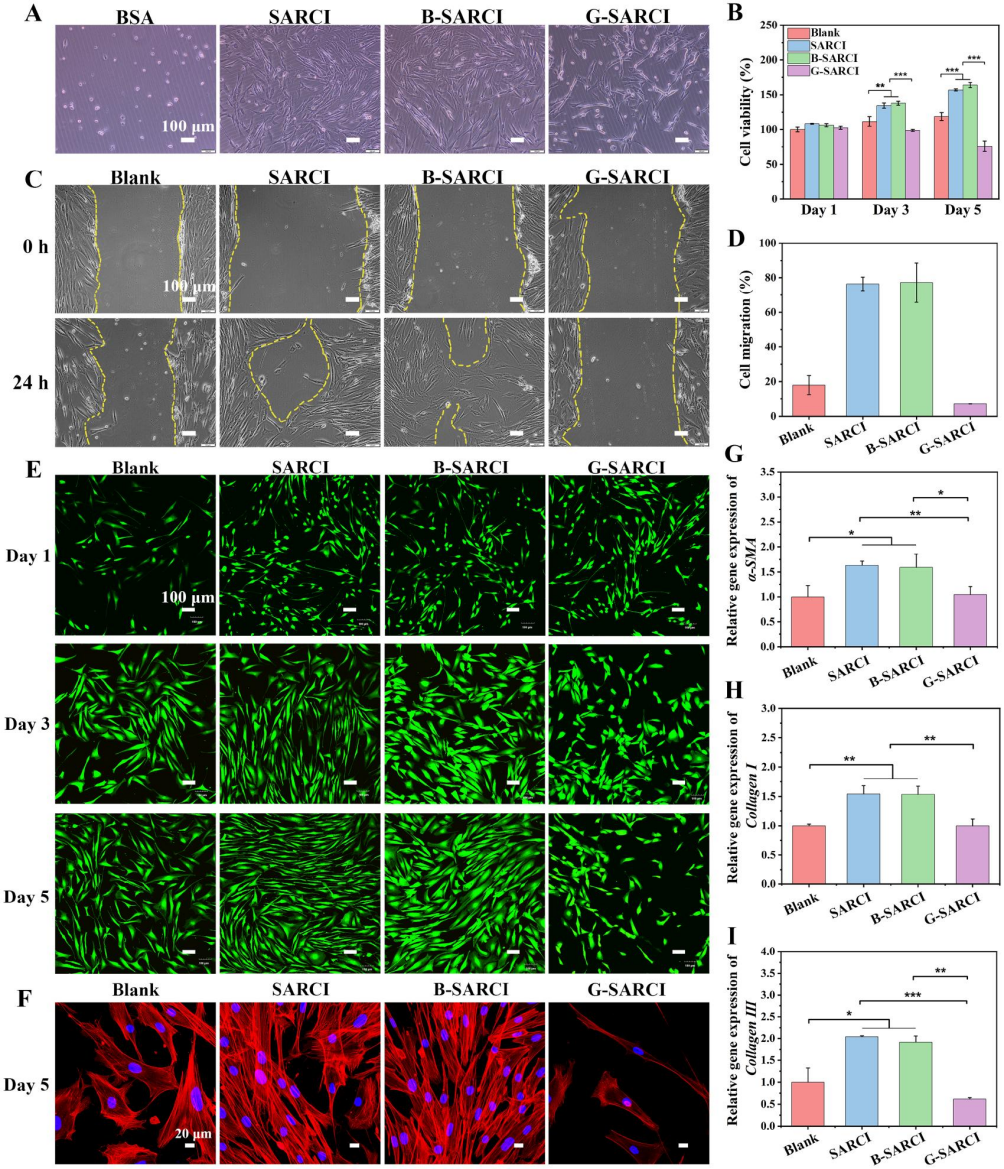
Evaluation of *in vitro* cytocompatibility and bioactivity of HFF-1 cells on B-SARCI. (**A**) Optical micrographs displaying cellular attachment and morphology after 12 h of culture. (**B**) Quantification of cell proliferative activity using the CCK-8 kit. (**C**) Cellular motility investigated via the scratch assay after a 24 h interval. (**D**) Wound closure rates. (**E**) Live/dead fluorescence staining. (**F**) Cytoskeletal architecture revealed by phalloidin (F-actin, red) and hoechst (nuclei, blue). (**G**-**I**) RT-qPCR-based expression signatures for *α-SMA*, *collagen I*, and *collagen III*. Quantitative results are illustrated as mean ± SD. *P < 0.05; **P < 0.01; ***P < 0.001.

To assess the biological performance and cellular interactions of B-SARCI, assays were conducted using human foreskin fibroblasts (HFF-1), with SARCI, G-SARCI, and bovine serum albumin (BSA) serving as controls (Figure 3). Cell adhesion assays revealed distinct differences in cell-substrate interactions. HFF-1 cells on BSA-coated surfaces remained round and poorly attached, whereas those cultured on SARCI exhibited extensive spreading and elongated morphologies, indicative of favorable substrate affinity and well-organized cytoskeletal structure. In contrast, G-SARCI supported only limited cell spreading, likely due to surface cytotoxicity and reduced bioactivity resulting from residual aldehyde groups. B-SARCI, however, promoted the most extensive cell spreading and elongation, reflecting superior substrate compatibility and enhanced adhesion (Figure 3A). After five days of culture, phalloidin staining revealed well-organized actin structures with more coherent cytoskeletal organization in cells on B-SARCI coatings, which was more pronounced than that on G-SARCI, supporting the notion that B-SARCI provides a favorable microenvironment for fibroblast attachment and cytoskeletal organization (Figure 3F).

The proliferative activity of HFF-1 fibroblasts cultured on different collagen matrices was quantified using the CCK-8 assay. Cell proliferation progressively increased in all groups except G-SARCI, which showed a time-dependent decline in viability (102.3, 98.7, and 76.0%). In contrast, SARCI (107.9, 134.3, and 156.9%) and particularly B-SARCI (105.9, 137.7, and 163.8%) showed higher cell viability than the blank group (100, 111.6, and 118.7%) across the time points (Figure 3B). Live/dead fluorescence staining further corroborated the quantitative results, revealing densely distributed viable cells with minimal cell death in the B-SARCI group, thereby confirming its excellent cytocompatibility and negligible cytotoxicity (Figure 3E).

The pro-migratory capacity of B-SARCI was further examined using a scratch-wound assay (Figure 3C, D). Both SARCI and B-SARCI significantly accelerated wound closure, reaching migration rates of 76.4 and 77.2% after 24 h, respectively, compared with minimal migration in the untreated control and only 7.1% in the G-SARCI group. These findings indicate that B-SARCI effectively enhances fibroblast motility, thereby facilitating cellular processes that are fundamental to tissue repair and regenerative remodeling.

To assess the differentiation-promoting ability of the scaffolds, the expression levels of fibroblast-specific genes were quantified using real-time quantitative polymerase chain reaction (RT-qPCR). *α-SMA*, a marker of fibroblast-to-myofibroblast transition, was significantly upregulated, with expression levels increased by 1.63-fold and 1.59-fold in the SARCI and B-SARCI groups, respectively (Figure 3G). Type I and type III collagens, the principal structural components of the extracellular matrix, were also markedly elevated. Specifically, *Collagen I* expression increased 1.54-fold in the SARCI group and 1.53-fold in the B-SARCI group, whereas *Collagen III* expression rose 2.03-fold and 1.91-fold, respectively. In contrast, G-SARCI did not elicit comparable gene upregulation (Figure 3H and I). The progressive decline in HFF-1 viability in the G-SARCI group suggests reduced cytocompatibility that may contribute to the attenuated transcriptional responses. Residual glutaraldehyde or unreacted aldehyde groups in GA-crosslinked collagenous matrices have been reported to impair cytocompatibility and could represent one plausible contributing factor [27]. These results confirm the favorable bioactivity of B-SARCI in enhancing fibroblast differentiation and extracellular matrix synthesis, which are key processes in dermal remodeling and tissue regeneration.

Collectively, these findings demonstrated that B-SARCI facilitated robust cell-material interactions, effectively promoting adhesion, proliferation, and migration, while also enhancing fibroblast differentiation. This multifunctional bioactivity highlighted its promise as a versatile platform for skin regeneration.

### *In vivo* biocompatibility and immunomodulation of B-SARCI

**Figure 4:**
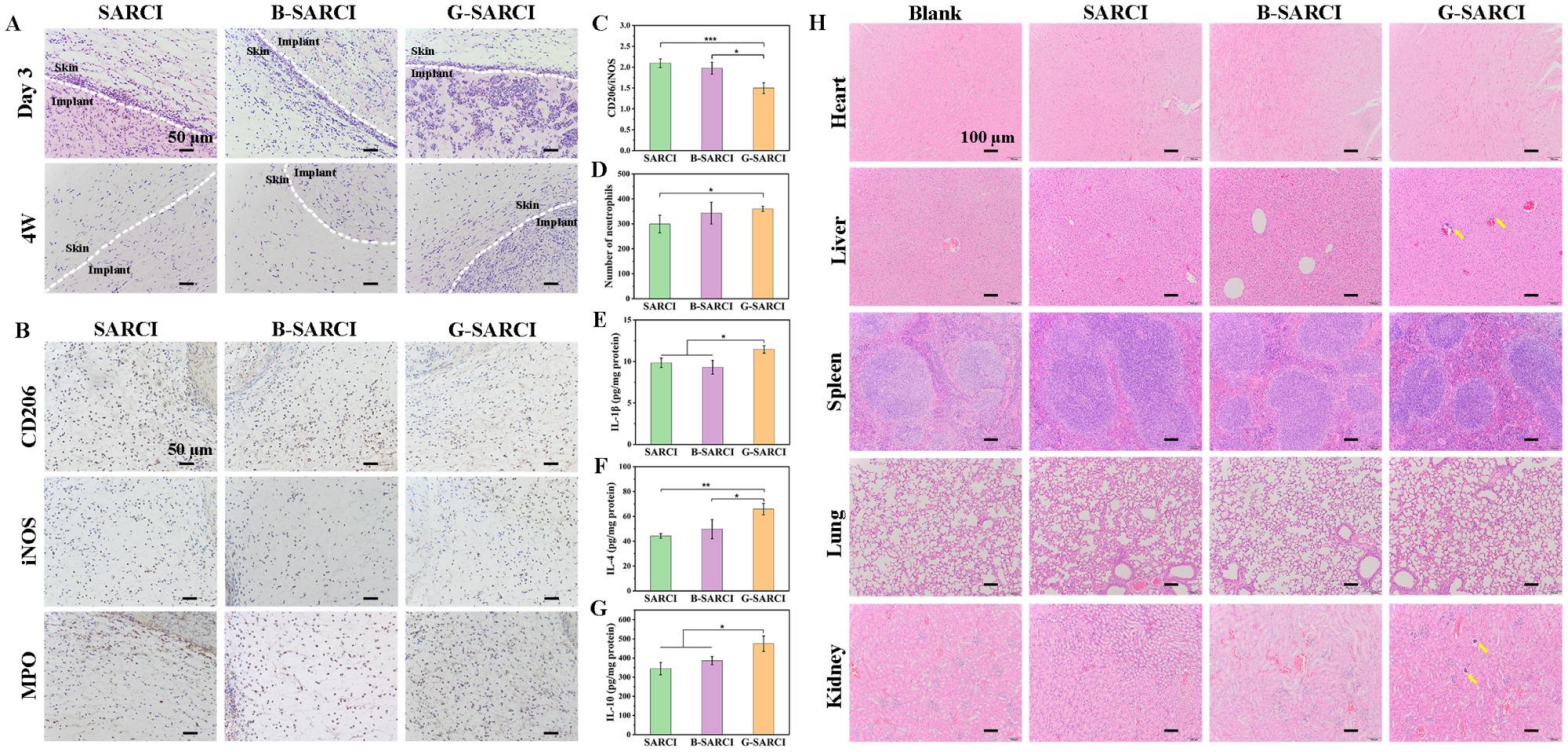
*In vivo* biocompatibility and immunomodulatory evaluation of recombinant collagen implants. (**A**) Representative H&E staining of implanted sites at day 3 and week 4 post-implantation. The skin-implant interface is indicated by the white line. (**B**) Representative immunohistochemical images of CD206, iNOS, and MPO at day 3. (**C**) Quantification of the CD206/iNOS ratio indicating macrophage polarization balance. (**D**) Quantification of MPO-positive neutrophils. ELISA quantification of (**E**) IL-1β, (**F**) IL-4, and (**G**) IL-10 at week 4. (**H**) Representative H&E-stained sections of the heart, liver, spleen, lung, and kidney harvested at week 6. Yellow arrows denote inflammatory cell infiltration. Quantitative results are illustrated as mean ± SD. *P < 0.05; **P < 0.01; ***P < 0.001.

Histological and biochemical analyses were conducted to evaluate the local and systemic biocompatibility of the collagen implants. Dense inflammatory infiltrates at the implant-tissue interface in all groups (SARCI, B-SARCI, and G-SARCI) on day 3, indicating an acute inflammatory response. Such acute inflammation is a typical early host response after implantation and constitutes the initial stage of the foreign body reaction to biomaterials [28]. The inflammatory response had largely subsided in both the SARCI and B-SARCI groups by week 4, whereas notable inflammatory cell accumulation remained at the implant boundary in the G-SARCI group, indicating delayed resolution of inflammation (Figure 4A). The day 3 and week 4 time points were selected to capture distinct phases of host-material interactions, with day 3 reflecting early innate immune cell infiltration and inflammatory activity and week 4 corresponding to the period of inflammation resolution and tissue remodeling routinely monitored in biomaterial research [29, 30].

To characterize macrophage phenotypes, immunohistochemical staining for iNOS (M1) and CD206 (M2) was performed. Notably, the SARCI and B-SARCI groups exhibited a markedly higher CD206/iNOS ratio compared to the G-SARCI group (Figure 4B and C), demonstrating a predominance of anti-inflammatory M2 macrophages and a microenvironment favorable for regeneration [31]. Myeloperoxidase (MPO), a neutrophil-associated enzyme marking early inflammation, was markedly elevated in G-SARCI compared with SARCI and B-SARCI (Figure 4B and D) [32]. The secretion of immune modulators (IL-1β, IL-4, and IL-10) is triggered by neutrophils undergoing frustrated phagocytosis when faced with persistent xenobiotic challenges [33]. Enzyme-linked immunosorbent assay (ELISA) results revealed that these cytokines remained substantially higher in G-SARCI relative to SARCI and B-SARCI, confirming prolonged inflammatory activation (Figure 4E–G). Together, these findings indicate that B-SARCI elicits a milder and self-resolving immune response characterized by attenuated neutrophil activity and M2-biased macrophage polarization, whereas G-SARCI induces sustained inflammation.

Major organs (heart, liver, spleen, lung, and kidney) were harvested and stained with H&E to evaluate potential systemic toxicity (Figure 4H). The systemic safety of both SARCI and B-SARCI was confirmed by the lack of discernible histopathological lesions across major organs, indicating an absence of adverse physiological impact. Conversely, the G-SARCI group revealed inflammatory lesions in the liver and kidneys, indicating potential systemic toxic effects associated with the GA-crosslinked control. Collectively, these results confirm the superior *in vivo* biocompatibility and immunomodulatory capacity of B-SARCI compared with conventional glutaraldehyde-crosslinked collagen implants, supporting its safety and translational potential for clinical skin-regeneration applications.

### Restorative effects of B-SARCI in a UV-driven skin photoaging model

**Figure 5:**
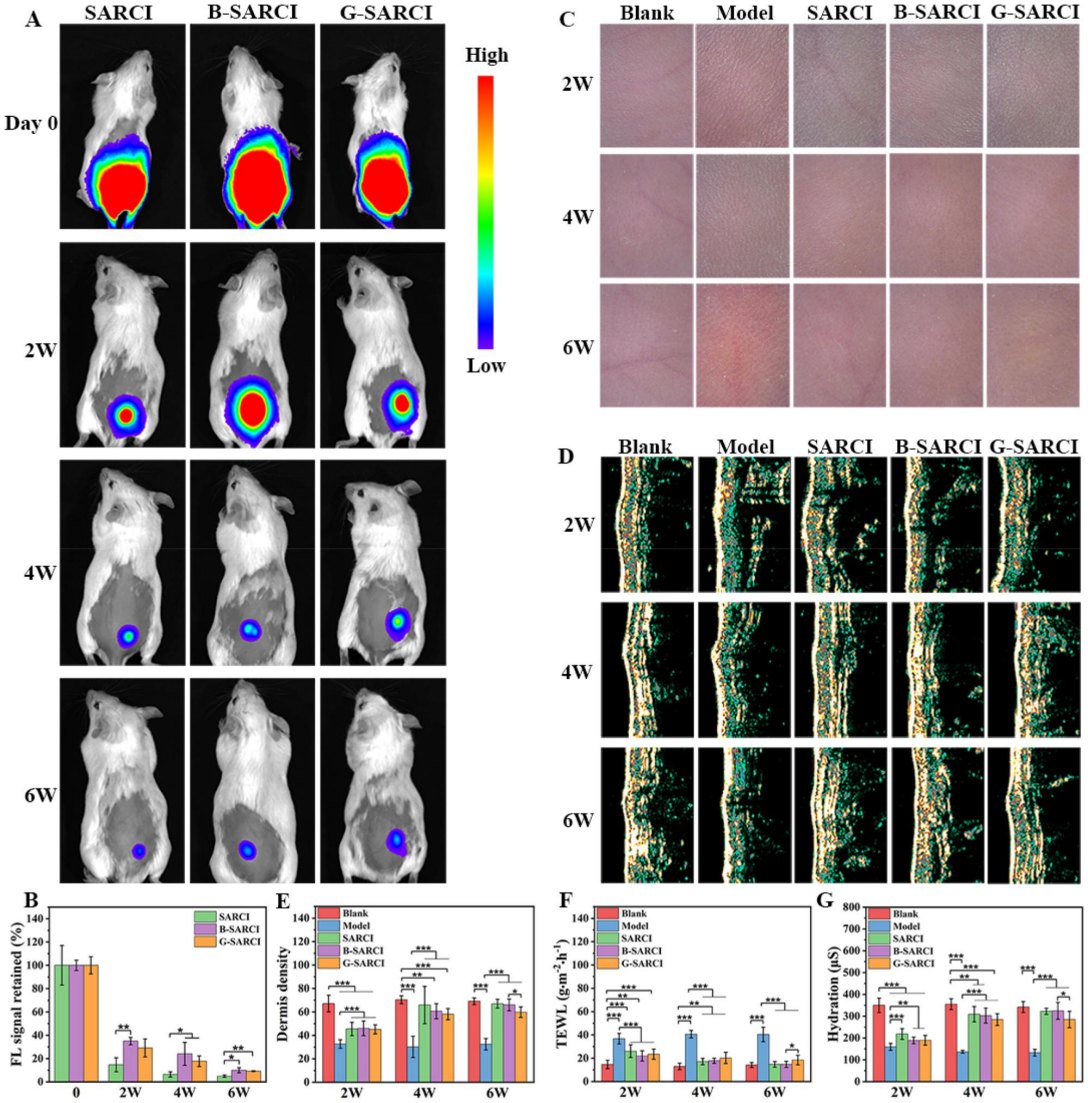
Restorative effects of B-SARCI against UV-driven skin photoaging. (**A**) Representative NIR fluorescence images of the implants *in vivo*. (**B**) Quantitative analysis of *in vivo* degradation based on fluorescence intensity. For each group, the signal on day 0 was normalized to 100%. (**C**) Representative dermoscopy illustrating the evolution of skin texture and wrinkling. (**D**) High-frequency ultrasonic imaging. (**E**) Dermal density quantification. (**F**) TEWL and (**G**) skin hydration were recorded at the specified intervals. Quantitative results are illustrated as mean ± SD. *P < 0.05; **P < 0.01; ***P < 0.001.

To investigate the *in vivo* persistence, capacity for ECM remodeling, and anti-photoaging effects of B-SARCI, a mouse model of UV-driven photoaging was employed (Figure 5). Near-infrared (NIR) fluorescence imaging was performed to further monitor *in vivo* degradation of the implants. B-SARCI exhibited significantly delayed *in vivo* degradation compared with SARCI. Notably, the degradation kinetics of G-SARCI were comparable to those of B-SARCI, with no statistically significant difference observed between the two groups (Figure 5A and B). To determine whether the week-6 residual lesion represented an abscess, H&E staining was conducted. No evident abscess-like lesions were observed in the SARCI or B-SARCI groups, whereas dense inflammatory cell infiltration persisted at the implant-tissue interface in the G-SARCI group (Figure S3A). In addition, CD68 immunofluorescence staining at weeks 2, 4, and 6 with quantitative analysis showed a progressive decrease in CD68-positive area over time for SARCI and B-SARCI groups, suggesting attenuation of macrophage-associated inflammation during implant degradation. In contrast, the G-SARCI group maintained a relatively high CD68-positive area, indicating a more sustained macrophage response (Figure S3B, C). The difference may be more closely related to GA-specific chemical cues, such as residual glutaraldehyde or unreacted aldehyde functionalities, which have been reported to adversely affect local cytocompatibility and inflammatory responses [34, 35]. These findings suggest that BDDE crosslinking enhances *in vivo* implant stability while eliciting a milder local inflammatory response, which may contribute to the sustained therapeutic performance of B-SARCI in photoaged skin.

Dermoscopy was used to monitor surface morphology and wrinkle formation throughout the study (Figure 5C). Two weeks post-irradiation, all groups exhibited evident wrinkling and dryness, most prominently in the model and G-SARCI groups. By week 6, skin treated with SARCI or B-SARCI appeared smoother and nearly wrinkle-free, whereas the model group displayed deepened wrinkles and coarse texture. G-SARCI treatment yielded partial improvement but remained inferior to SARCI and B-SARCI. These results demonstrate that B-SARCI effectively mitigates wrinkle formation and restores skin smoothness following UV-induced damage.

High-frequency ultrasound imaging revealed progressive structural recovery of the dermis (Figure 5D and E). At weeks 2 and 4, signal intensity analysis showed that all treated groups exhibited higher dermal density than the model group. By week 6, the SARCI and B-SARCI groups exhibited substantial tissue recovery, with dermal density returning to near-baseline values, whereas the G-SARCI group remained significantly below normal. These findings underscore the superior ability of B-SARCI to restore dermal architecture and extracellular matrix organization.

To monitor the restoration of the skin barrier, transepidermal water loss (TEWL) levels were measured (Figure 5F). UV irradiation induced a sharp increase in TEWL in all UV-exposed groups, reflecting barrier disruption. During the treatment period, TEWL values in the SARCI and B-SARCI groups underwent a steady decrease and successfully equilibrated with the baseline values at the 6-week assessment point, whereas the model group retained high TEWL throughout. The G-SARCI group exhibited only partial recovery, indicating incomplete restoration of barrier function. Correspondingly, skin hydration dropped markedly at week 2 in all UV-exposed groups. At the final time point, hydration recovered in the SARCI and B-SARCI groups, whereas the model and G-SARCI groups exhibited sustained dehydration (Figure 5G).

Together, the evidence suggests that B-SARCI not only enhances implant stability but also effectively restores dermal density, barrier integrity, and hydration, leading to visible improvement in skin texture and elasticity. These outcomes highlight B-SARCI as a potent injectable biomaterial for reversing UV-induced skin photoaging and re-establishing cutaneous homeostasis.

**Figure 6:**
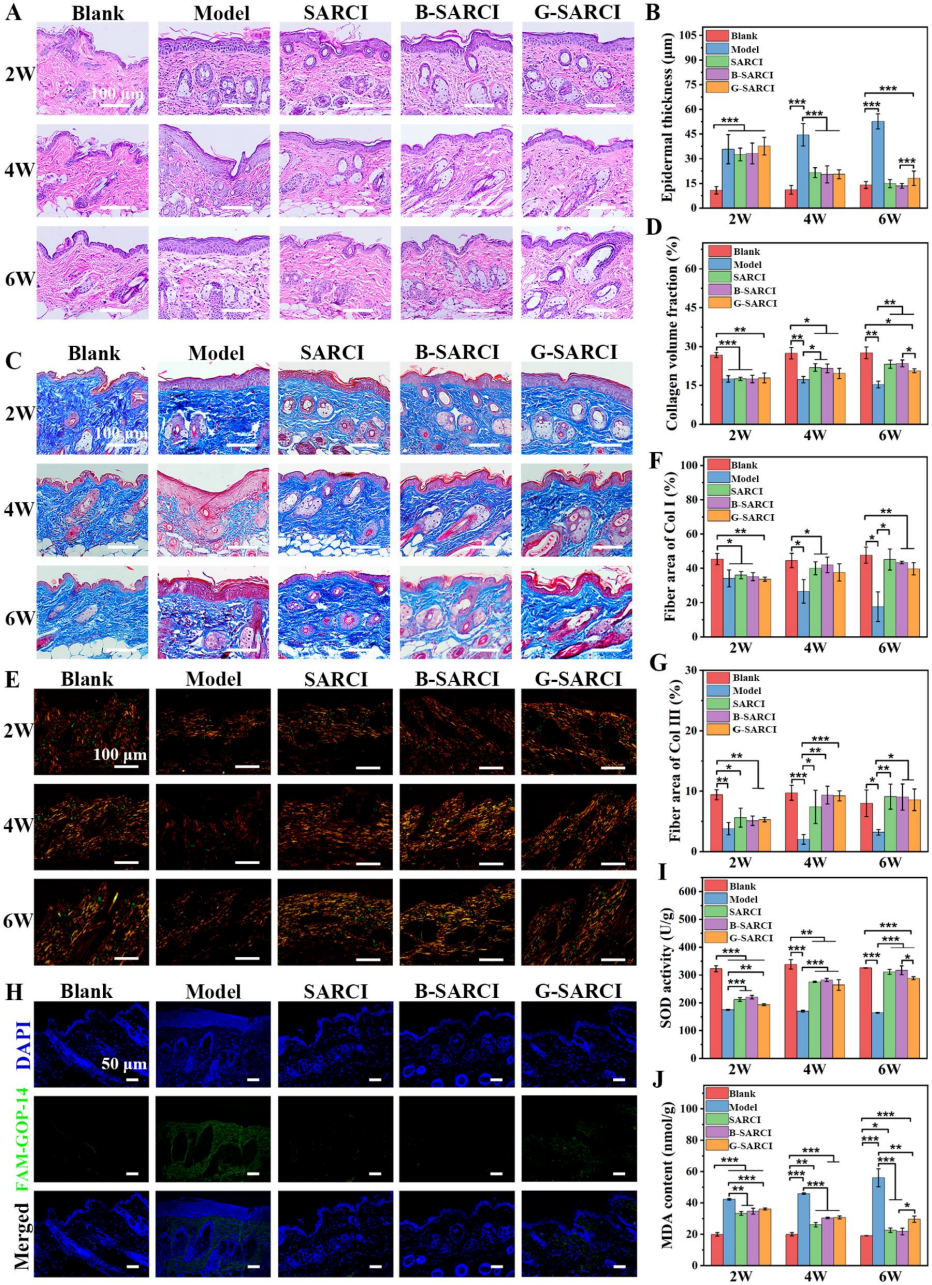
Morphological and biochemical assessment of skin tissue repair post-B-SARCI intervention. (**A**) Representative H&E histology. (**B**) Epidermal thickness. (**C**) Masson’s trichrome staining. (**D**) Cermal collagen volume fraction. (**E**) Polarized-light images after picrosirius red staining. (**F**) Col I and (**G**) Col III levels. (**H**) Confocal imaging with the FAM-GOP-14 probe. (**I**) SOD activity. (**J**) MDA content. Quantitative results are illustrated as mean ± SD . *P < 0.05; **P < 0.01; ***P < 0.001.

Histological examination was performed to evaluate the structural restoration mediated by B-SARCI (Figure 6A and B). At week 2, H&E displayed marked epidermal thickening throughout the UV-treated groups. This hyperplastic response persisted in the untreated model group, whereas SARCI- and B-SARCI-treated skin showed gradual normalization of epidermal architecture, approaching baseline morphology by week 6. Although G-SARCI induced partial recovery, epidermal thickness remained significantly greater relative to the healthy control. This indicates that B-SARCI effectively preserves epidermal homeostasis and mitigates photoaging-associated hyperplasia.

The regenerative progress of dermal collagen was investigated through Masson’s trichrome staining (Figure 6C and D). At week 2, a pronounced depletion of dermal collagen was observed in all UV-irradiated groups relative to the healthy control. Collagen degradation continued in the model group, whereas SARCI and B-SARCI interventions facilitated a continuous restoration of collagen deposition, reaching near-normal levels by week 6. G-SARCI supported only a limited recovery of collagen levels, which remained below those of the healthy control group. These findings highlight the greater potential of B-SARCI in promoting dermal collagen remodeling and matrix reconstitution.

Collagen subtype architecture was examined via Picrosirius red staining. Red/yellow and green signals were indicative of Type I and Type III collagen fibers, respectively (Figure 6E–G). UV exposure induced a significant degradation of both collagen species two weeks post-irradiation. Collagen loss progressed further with time for model group, whereas SARCI-and particularly B-SARCI-induced steady recovery of collagen I and III, approaching healthy levels at the final assessment. In the G-SARCI group, however, type I collagen remained significantly reduced, underscoring the more robust matrix-restorative capacity of B-SARCI.

FAM-GOP-14, a specialized peptide, was employed to target unfolded collagen. Its selectivity relies on recognizing disrupted triple-helices, with negligible interaction observed in the presence of native triple-helical collagen [36]. At the final assessment point, marked fluorescent signals permeated the dermis of the model subjects, signifying the vast accumulation of misfolded collagen. Conversely, fluorescent signals were barely discernible within the three groups (blank, SARCI, and B-SARCI), suggesting maintenance of the native collagen structure. Moderate fluorescence in the G-SARCI group indicated partial collagen damage (Figure 6H). These results demonstrate that B-SARCI effectively maintains collagen structural integrity and facilitates organized dermal repair.

The antioxidant capacity and lipid peroxidation were measured to assess redox homeostasis (Figure 6I and J). Superoxide dismutase (SOD) activity reflects endogenous antioxidant defense capacity, whereas malondialdehyde (MDA) is a terminal byproduct of lipid peroxidation and is widely used as a biomarker of oxidative damage associated with lipid peroxidation and oxidative stress [37, 38]. Substantial oxidative stress was evident at the two-week time point, as evidenced by the diminished SOD activity and augmented MDA levels across all irradiated groups relative to the blank control. While redox imbalance exacerbated throughout the study in the untreated group, SARCI and B-SARCI interventions promoted a steady normalization of antioxidant defenses and lipid peroxidation, nearly returning to baseline by the 6-week mark (Figure 6I and J). These findings indicate that B-SARCI exerts potent antioxidative effects, promoting recovery of cellular redox homeostasis during skin repair.

In summary, B-SARCI offered a comprehensive rescue of photoaged skin by normalizing epidermal thickness, replenishing collagen density, and reinforcing antioxidant defenses. NIR fluorescence monitoring further confirmed its prolonged *in vivo* retention, supporting improved durability under physiological conditions. This enhanced persistence may be particularly relevant for applications requiring sustained structural support or extended local bioactivity. However, whether it translates into superior long-term functional outcomes will require longer follow-up and additional endpoints.

### Mechanistic insights into B-SARCI-enabled recovery of photoaged skin

**Figure 7:**
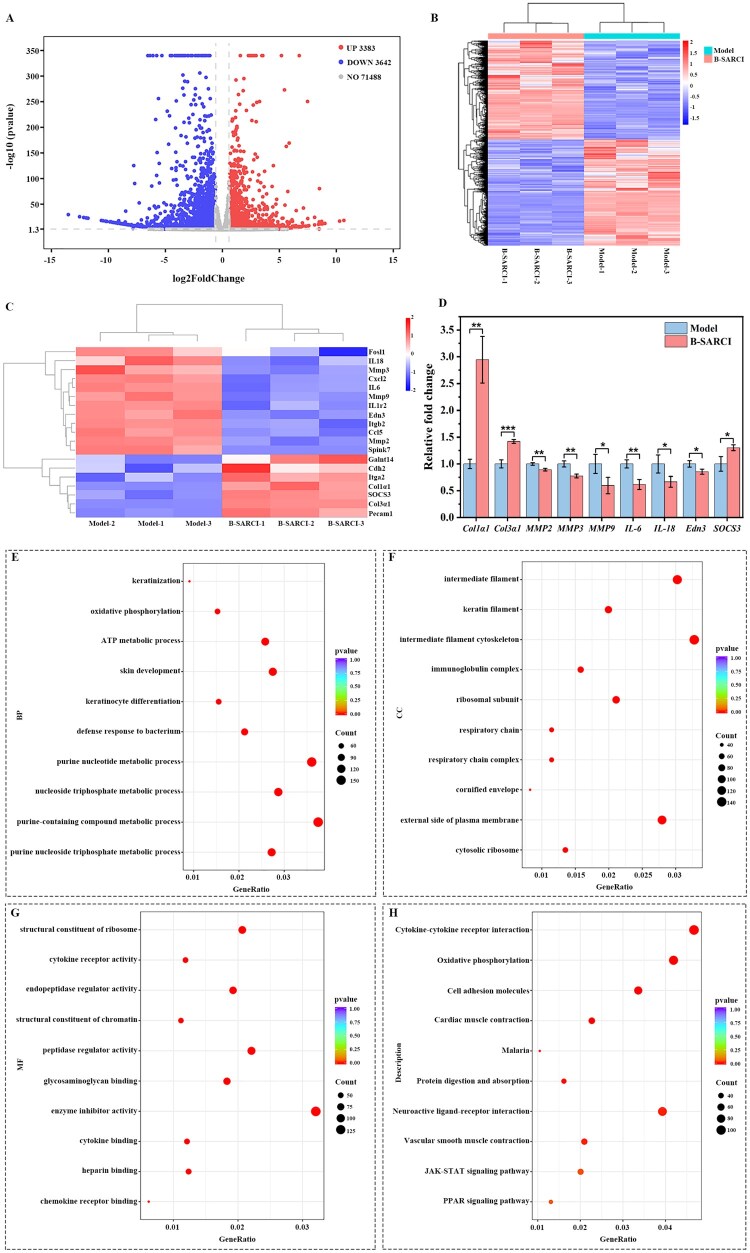
Transcriptomic and molecular assessment of B-SARCI-mediated skin tissues. (**A**) Volcano plot. (**B**) Hierarchical clustering of DEGs. (**C**) Heatmap of representative ECM-related and inflammation-associated genes. (**D**) RT-qPCR validation of selected gene expression profiles. (**E**-**G**) GO enrichment analyses highlighting biological processes (BP), cellular components (CC), and molecular functions (MF) involved in tissue repair. (**H**) KEGG pathway enrichment analysis. Quantitative results are illustrated as mean ± SD. *P < 0.05; **P < 0.01; ***P < 0.001.

Transcriptomic analysis was utilized to investigate the regulatory mechanisms of B-SARCI in skin repair (Figure 7). Volcano plot and heatmap-based clustering identified a global shift in gene expression profiles post-treatment. Specifically, B-SARCI intervention led to the elevation of 3383 genes and the suppression of 3642 others compared to the model group (Figure 7A and B). Functional enrichment analysis showed that differentially expressed genes (DEGs) were largely involved in modulating inflammatory responses and ECM remodeling. Representative DEGs included matrix-associated genes (*Col1α1*, *Col3α1*, *MMP2*, *MMP3,* and *MMP9*) and cytokine receptors (*IL-6* and *IL-18*), highlighting B-SARCI’s dual role in structural repair and immune modulation (Figure 7C).

Gene Ontology (GO) enrichment analysis revealed that B-SARCI treatment activated biological processes related to keratinization, cellular differentiation, protein synthesis, energy metabolism, and immune defense, while maintaining cytoskeletal organization and enzymatic balance-reflecting an integrated state of dermal remodeling and barrier restoration **(**Figure 7E–G**)**. Kyoto Encyclopedia of Genes and Genomes (KEGG) enrichment further revealed coordinated regulation of multiple signaling cascades, including inflammatory response attenuation, cell-matrix interaction enhancement, and lipid homeostasis recovery. Notably, upregulation of *SOCS3* and concurrent downregulation of *IL-6* within the JAK-STAT pathway may indicate a transcriptional feedback mechanism that limits excessive inflammation and promotes tissue equilibrium (Figure 7H).

To validate key transcriptomic findings, RT-qPCR was used to quantify mRNA levels of selected ECM-remodeling and inflammation-related genes (Figure 7D). The structural collagens *Col1α1* and *Col3α1*, essential for dermal integrity and elasticity, were expressed at significantly higher levels in the B-SARCI group. In contrast, expression of matrix metalloproteinases *MMP2*, *MMP3*, and *MMP9* was markedly downregulated, suggesting suppression of ECM degradation and stabilization of dermal structure. Moreover, B-SARCI treatment decreased *IL-6* expression while enhancing *SOCS3*, corroborating its regulatory effect on the JAK-STAT pathway to mitigate inflammation.

Collectively, these findings reveal that B-SARCI orchestrates a multifaceted repair mechanism involving enhanced collagen biosynthesis, reduced matrix degradation, and attenuation of inflammatory signaling. This integrated molecular response underlies its ability to restore structural integrity, promote dermal remodeling, and re-establish homeostasis in photoaged skin.

**Figure 8:**
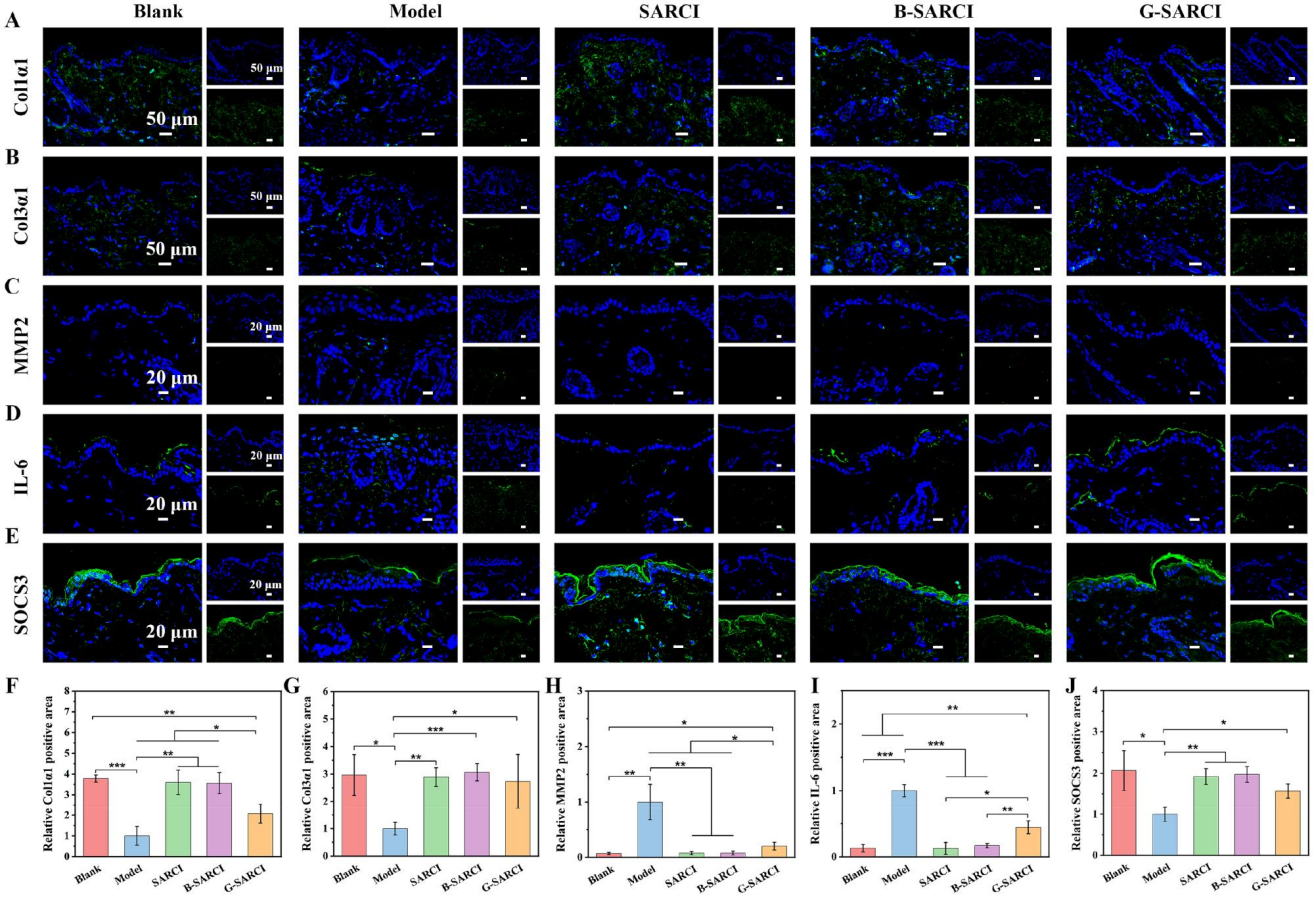
(**A**-**E**) Typical images of skin sections stained for Col1α1, Col3α1, MMP2, IL-6, and SOCS3. (**F**-**J**) Corresponding quantification of the positive area for the indicated extracellular matrix and inflammatory markers. The positive area was normalized to the model group (set to 1). Quantitative results are illustrated as mean ± SD. *P < 0.05; **P < 0.01; ***P < 0.001.

The expression of key extracellular matrix components and regulatory enzymes were assessed via immunofluorescence staining (Figure 8). In contrast to the untreated model, SARCI, B-SARCI, and G-SARCI treatments effectively promoted Col1α1 and Col3α1 production and down-regulated the expression of MMP2. Notably, SARCI and B-SARCI produced more pronounced Col1α1 accumulation and MMP2 inhibition than G-SARCI, suggesting a greater capacity for extracellular matrix remodeling. In addition, the treatments differed in their modulation of inflammatory markers. The SARCI and B-SARCI groups showed a marked reduction in IL-6 accompanied by an increase in SOCS3 relative to the model group, whereas G-SARCI-treated skin exhibited higher IL-6 levels and weaker SOCS3 induction than the SARCI and B-SARCI groups, suggesting a less effective attenuation of inflammatory signaling [39]. These results indicate that B-SARCI simultaneously promotes type I and III collagen synthesis, inhibits MMP-mediated matrix degradation, and modulates IL-6/SOCS3 signaling in a manner consistent with attenuation of JAK-STAT-associated inflammatory activation, thereby facilitating structural reconstruction and functional recovery of photoaged skin.

## Conclusion

Photoaging, a prototypical form of extrinsic skin aging arising from cumulative UV exposure, induces progressive collagen depletion, matrix metalloproteinase activation, and extracellular matrix (ECM) disorganization, ultimately compromising dermal architecture and function. Although animal-derived collagens have long served as structural biomaterials, their intrinsic immunogenicity, compositional variability, and pathogen risks constrain reproducible clinical translation. Recombinant collagen offers a biosafe alternative with molecularly defined sequences, yet its insufficient fibrillogenesis and poor *in vivo* persistence continue to impede therapeutic efficacy.

Here, we engineered an injectable recombinant collagen nanofiber implant (B-SARCI) through the synergistic coupling of molecular self-assembly and mild BDDE crosslinking. This dual design paradigm endows higher thermal and enzymatic stability, along with excellent syringeability. B-SARCI established a bioactive microenvironment that enhanced fibroblast adhesion, migration, proliferation, and differentiation, confirming its capacity to recapitulate ECM cues and drive dermal cell functionality.

In a mouse model of UV-driven photoaging, B-SARCI significantly re-established dermal density, normalized epidermal structure, and improved barrier performance. Transcriptomic and histological analyses revealed that B-SARCI reprogrammed the dermal transcriptome toward matrix reconstruction and inflammation resolution, characterized by upregulation of Col1α1/Col3α1, suppression of MMP2/3/9, and modulation of the JAK-STAT pathway via SOCS3 induction and IL-6 attenuation. These findings delineate a coordinated molecular mechanism through which B-SARCI reinstates ECM homeostasis and mitigates chronic photoaging-associated inflammation.

Collectively, this work establishes a next-generation paradigm for recombinant collagen biomaterials-wherein controlled self-assembly and biocompatible crosslinking converge to yield a durable, injectable, and intrinsically bioactive implant. By bridging molecular precision with functional tissue regeneration, B-SARCI exemplifies a clinically translatable strategy for dermal reconstruction and aesthetic skin rejuvenation.

## Supplementary Material

rbag026_Supplementary_Data

## Data Availability

Data will be made available on request.

## References

[1] Thau H , Gerjol BP, Hahn K, Gudenberg RW, Knoedler L, Stallcup K, Emmert MY, Buhl T, Wyles SP, Tchkonia T, Tullius SG, Iske J. Senescence as a molecular target in skin aging and disease. Ageing Res Rev 2025;105:102686. 39929368 10.1016/j.arr.2025.102686

[2] Franco AC , Aveleira C, Cavadas C. Skin senescence: mechanisms and impact on whole-body aging. Trends Mol Med 2022;28:97–109. 35012887 10.1016/j.molmed.2021.12.003

[3] Tang X , Yang T, Yu D, Xiong H, Zhang S. Current insights and future perspectives of ultraviolet radiation (UV) exposure: friends and foes to the skin and beyond the skin. Environ Int 2024;185:108535. 38428192 10.1016/j.envint.2024.108535

[4] Ke Y , Wang XJ. TGFβ signaling in photoaging and UV-induced skin cancer. J Invest Dermatol 2021;141:1104–10. 33358021 10.1016/j.jid.2020.11.007 PMC7987776

[5] Kim H , Jang J, Song MJ, Park CH, Lee DH, Lee SH, Chung JH. Inhibition of matrix metalloproteinase expression by selective clearing of senescent dermal fibroblasts attenuates ultraviolet-induced photoaging. Biomed Pharmacother 2022;150:113034. 35489284 10.1016/j.biopha.2022.113034

[6] Choi JS , Cho WL, Choi YJ, Kim JD, Park HA, Kim SY, Park JH, Jo DG, Cho YW. Functional recovery in photo-damaged human dermal fibroblasts by human adipose-derived stem cell extracellular vesicles. J Extracell Vesicles 2019;8:1565885. 30719241 10.1080/20013078.2019.1565885 PMC6346706

[7] Oppel T , Korting HC. Actinic keratosis: the key event in the evolution from photoaged skin to squamous cell carcinoma: therapy based on pathogenetic and clinical aspects. Skin Pharmacol Physiol 2004;17:67–76. 14976383 10.1159/000076016

[8] Yammine KM , Li RC, Borgula IM, Abularach SM, DiChiara AS, Raines RT, Shoulders MD. An outcome-defining role for the triple-helical domain in regulating collagen-I assembly. Proc Natl Acad Sci U S A 2024;121:e2412948121. 39503893 10.1073/pnas.2412948121 PMC11573663

[9] Yu LT , Hancu MC, Kreutzberger MAB, Henrickson A, Demeler B, Egelman EH, Hartgerink JD. Hollow octadecameric self-assembly of collagen-like peptides. J Am Chem Soc 2023;145:5285–96. 36812303 10.1021/jacs.2c12931 PMC10131286

[10] Kirkness MW , Lehmann K, Forde NR. Mechanics and structural stability of the collagen triple helix. Curr Opin Chem Biol 2019;53:98–105. 31606538 10.1016/j.cbpa.2019.08.001

[11] Lin K , Zhang D, Macedo MH, Cui W, Sarmento B, Shen G. Advanced collagen-based biomaterials for regenerative biomedicine. Adv Func Mater 2019;29:1804943.

[12] Chang S , Zhao M, Gao W, Cao J, He B. Engineered collagen/PLLA composite fillers to induce rapid and long-term collagen regeneration. J Mater Chem B 2025;13:904–17. 39659187 10.1039/d4tb02159b

[13] Wang Q , Yan H, Yao L, Li W, Xiao J. A highly biocompatible CE-crosslinked collagen implant with exceptional anti-calcification and collagen regeneration capabilities for aging skin rejuvenation. J Mater Chem B 2024;12:4467–77. 38629894 10.1039/d3tb03032f

[14] Meganathan I , Sundarapandian A, Shanmugam G, Ayyadurai N. Three-dimensional tailor-made collagen-like proteins hydrogel for tissue engineering applications. Biomater Adv 2022;139:212997. 35882145 10.1016/j.bioadv.2022.212997

[15] Cao L , Zhang Z, Yuan D, Yu M, Min J. Tissue engineering applications of recombinant human collagen: a review of recent progress. Front Bioeng Biotechnol 2024;12:1358246. 38419725 10.3389/fbioe.2024.1358246 PMC10900516

[16] Guo X , Ma Y, Wang H, Yin H, Shi X, Chen Y, Gao G, Sun L, Wang J, Wang Y, Fan D. Status and developmental trends in recombinant collagen preparation technology. Regen Biomater 2024;11:rbad106. 38173768 10.1093/rb/rbad106 PMC10761200

[17] Meganathan I , Pachaiyappan M, Aarthy M, Radhakrishnan J, Mukherjee S, Shanmugam G, You J, Ayyadurai N. Recombinant and genetic code expanded collagen-like protein as a tailorable biomaterial. Mater Horiz 2022;9:2698–721. 36189465 10.1039/d2mh00652a

[18] Guo J , Li H, Wang F. Orthogonal design-driven in situ encapsulation of hyaluronic acid-poly (lactic acid) composite hydrogels: mechanically tunable dermal fillers with enhanced enzymatic resistance. RSC Adv 2025;15:31830–9. 40917617 10.1039/d5ra04259c PMC12409612

[19] Zeng Z , Wei N, Cai X, Xiao J. A magnetic bifunctional endotoxin removal nano-agent for the efficient elimination of endotoxins in recombinant protein preparation. Int J Biol Macromol 2025;311:143663. 40311983 10.1016/j.ijbiomac.2025.143663

[20] Krutmann J , Bouloc A, Sore G, Bernard BA, Passeron T. The skin aging exposome. J Dermatol Sci 2017;85:152–61. 27720464 10.1016/j.jdermsci.2016.09.015

[21] Wang Z , Chen H, Wang Y, Wu C, Ye T, Xia H, Huang R, Deng J, Li Z, Huang Y, Yang Y. Recombinant filaggrin-2 improves skin barrier function and attenuates ultraviolet B (UVB) irradiation-induced epidermal barrier disruption. Int J Biol Macromol 2024;281:136064. 39341309 10.1016/j.ijbiomac.2024.136064

[22] Micheels P , Porcello A, Bezzola T, Perrenoud D, Quinodoz P, Kalia Y, Allémann E, Laurent A, Jordan O. Clinical perspectives on the injectability of cross-linked hyaluronic acid dermal fillers: a standardized methodology for commercial product benchmarking with inter-injector assessments. Gels 2024;10:101. 38391431 10.3390/gels10020101 PMC10888303

[23] Zhao M , Chang S, Wang Y, Cao J, Pu Y, He B, Pan S. Porous PLLA microspheres dispersed in HA/collagen hydrogel as injectable facial fillers to enhance aesthetic effects. Regen Biomater 2025;12:rbaf049. 40556787 10.1093/rb/rbaf049 PMC12187068

[24] De Boulle K , Glogau R, Kono T, Nathan M, Tezel A, Roca-Martinez JX, Paliwal S, Stroumpoulis D. A review of the metabolism of 1,4-butanediol diglycidyl ether-crosslinked hyaluronic acid dermal fillers. Dermatol Surg 2013;39:1758–66. 23941624 10.1111/dsu.12301 PMC4264939

[25] Zöller K , To D, Bernkop-Schnürch A. Biomedical applications of functional hydrogels: innovative developments, relevant clinical trials and advanced products. Biomaterials 2025;312:122718. 39084097 10.1016/j.biomaterials.2024.122718

[26] Yang H. Establishing acceptable limits of residual DNA. PDA J Pharm Sci Technol 2013;67:155–63. 23569076 10.5731/pdajpst.2013.00910

[27] Islam MM , AbuSamra DB, Chivu A, Argueso P, Dohlman CH, Patra HK, Chodosh J, Gonzalez-Andrades M. Optimization of collagen chemical crosslinking to restore biocompatibility of tissue-engineered scaffolds. Pharmaceutics 2021;13:832. 34204956 10.3390/pharmaceutics13060832 PMC8229326

[28] Franz S , Rammelt S, Scharnweber D, Simon JC. Immune responses to implants: a review of the implications for the design of immunomodulatory biomaterials. Biomaterials 2011;32:6692–709. 21715002 10.1016/j.biomaterials.2011.05.078

[29] Jhunjhunwala S , Aresta-DaSilva S, Tang K, Alvarez D, Webber MJ, Tang BC, Lavin DM, Veiseh O, Doloff JC, Bose S, Vegas A, Ma M, Sahay G, Chiu A, Bader A, Langan E, Siebert S, Li J, Greiner DL, Newburger PE, von Andrian UH, Langer R, Anderson DG. Neutrophil responses to sterile implant materials. PLoS One 2015;10:e0137550. 26355958 10.1371/journal.pone.0137550 PMC4565661

[30] Anderson JM , Rodriguez A, Chang DT. Foreign body reaction to biomaterials. Semin Immunol 2008;20:86–100. 18162407 10.1016/j.smim.2007.11.004 PMC2327202

[31] Du Q , Zhang Y, Tang J, Fang W, Lei X, Zhang J, Mu B, Li Y, Zuo Y, Li Y. Smart pH-responsive conductive polyurethane fibers: doping strategies for advanced wound care. ACS Appl Mater Interfaces 2025;17:37657–78. 40522825 10.1021/acsami.5c07297

[32] Lin W , Chen H, Chen X, Guo C. The roles of neutrophil-derived myeloperoxidase (MPO) in diseases: the new progress. Antioxidants 2024;13:132. 38275657 10.3390/antiox13010132 PMC10812636

[33] Liu S , Song L, Huang S, Liu Z, Xu Y, Wang Z, Qiu H, Wang J, Chen Z, Xiao Y, Wang H, Zhu X, Zhang K, Zhang X, Lin H. Hydroxyapatite microspheres encapsulated within hybrid hydrogel promote skin regeneration through the activation of calcium signaling and motor protein pathway. Bioact Mater 2025;50:287–304. 40292340 10.1016/j.bioactmat.2025.04.002 PMC12022663

[34] Jiang Z , Wu Z, Deng D, Li J, Qi X, Song M, Liu Y, Wu Q, Xie X, Chen Z, Tang Z, Tang Z. Improved cytocompatibility and reduced calcification of glutaraldehyde-crosslinked bovine pericardium by modification with glutathione. Front Bioeng Biotechnol 2022;10:844010. 35662844 10.3389/fbioe.2022.844010 PMC9160462

[35] Ren Y , Alkildani S, Burckhardt K, Kowitsch A, Radenkovic M, Stojanovic S, Najman S, Jung O, Liu L, Barbeck M. The influence of different crosslinking agents onto the physical properties, integration behavior and immune response of collagen-based barrier membranes. Front Bioeng Biotechnol 2024;12:1506433. 39834629 10.3389/fbioe.2024.1506433 PMC11743487

[36] Qin X , Song C, Yao L, Cai X, Xiao J. A highly specific and versatile biochip for ultra-sensitive quantification of denatured collagen in assessing collagen quality. Anal Chem 2024;96:15640–7. 39231145 10.1021/acs.analchem.4c02883

[37] Schieber M , Chandel NS. ROS function in redox signaling and oxidative stress. Curr Biol 2014;24:R453–R462. 24845678 10.1016/j.cub.2014.03.034 PMC4055301

[38] Murphy MP , Bayir H, Belousov V, Chang CJ, Davies KJA, Davies MJ, Dick TP, Finkel T, Forman HJ, Janssen-Heininger Y, Gems D, Kagan VE, Kalyanaraman B, Larsson NG, Milne GL, Nystrom T, Poulsen HE, Radi R, Van Remmen H, Schumacker PT, Thornalley PJ, Toyokuni S, Winterbourn CC, Yin H, Halliwell B. Guidelines for measuring reactive oxygen species and oxidative damage in cells and *in vivo*. Nat Metab 2022;4:651–62. 35760871 10.1038/s42255-022-00591-z PMC9711940

[39] Babon JJ , Varghese LN, Nicola NA. Inhibition of IL-6 family cytokines by SOCS3. Semin Immunol 2014;26:13–9. 24418198 10.1016/j.smim.2013.12.004 PMC3970923

