# Supplementary material for: Synergistic self-assembly and crosslinking yield a durable, bioactive, and injectable recombinant collagen implant for photoaging therapy
Source: Regen Biomater. 2026 Mar 2;13:rbag026. doi: 10.1093/rb/rbag026 (PMC13037810; doi:10.1093/rb/rbag026)
Supplement: rbag026_Supplementary_Data [file rbag026_supplementary_data.zip › Supporting information-B-SARCI.doc]

Supporting information

Synergistic self-assembly and crosslinking yield a durable, bioactive, and injectable recombinant collagen implant for photoaging therapy

Nannan Wei,^a,b‡^ Yuchen Zhang,^a,b‡^ Xinyu Tian, ^a,b^ Linyan Yao,^b,c^ Jianxi Xiao^a,b*^

^a^ State Key Laboratory of Natural Product Chemistry, College of Chemistry and Chemical Engineering, Lanzhou University, Lanzhou 730000, P. R. China

^b^ Gansu Engineering Research Center of Medical Collagen, Lanzhou 730000, P. R. China

^c^ State Key Laboratory of Animal Disease Control and Prevention, College of Veterinary Medicine, Lanzhou University, Lanzhou 730000, P. R. China

^‡^Equal contribution.

*Corresponding Author.

***Email: xiaojx@lzu.edu.cn**

**Figure S1.** Characterization of recombinant type I collagen (RCI). (A) SDS-PAGE. (B) CD spectra.

**Figure S2.** Characterization of G-SARCI. (A) TNBS-based crosslinking degree measurement. (B) DSC analysis. (C) Representative SEM micrograph. (D) Extrusion force.

**Figure S3.** (A) Representative H&E staining of implantation sites at week 6 post-implantation. The skin-implant interface is indicated by the black line. (B) Representative CD68 immunofluorescence of tissue sections at weeks 2, 4, and 6. The interface is marked by a white line. (C) Quantification of the CD68-positive area. Quantitative results are illustrated as mean ± SD (triplicate independent experiments). **P < 0.01; ***P < 0.001.

**Table S1.** The amino acid sequence of RCI.

| Recombinant collagen | Sequence |
| --- | --- |
| RCI | MHHHHHHADEQEEKAKVRTELIQELAQGLGGFEKKNFPTLGDEDLDHTYMTKLLTYLQEREQAENSWRKRLLKGIQDHALDLVPRGPPGPPGPPGPPGPPGPPGPPGPPGPPGPPGERGPPGPQGARGLPGAPGQMGPRGLPGERGRPGAPGPAGARGEPGAPGSKGDTGAKGEPGPVGVQGPPGPAGEEGKRGARGEPGPTGPAGPKGSPGEAGRPGEAGLPGERGPPGPQGARGLPGAPGQMGPRGLPGERGRPGAPGPAGARGEPGAPGSKGDTGAKGEPGPVGVQGPPGPAGEEGKRGARGEPGPTGPAGPKGSPGEAGRPGEAGLPGPPGPPGPPGPPGPPGPPGPPGPPGPPGPP |

**Table S2.** Quality control of B-SARCI.

| Test item | Result |
| --- | --- |
| Residual BDDE | 1.35 ± 0.24 ppm |
| Endotoxin level | 0.28 ± 0.02 EU/mL |
| Residual DNA content | 4.27 ± 0.58 ng/mL |
